# Precision Oncology for High-Grade Gliomas: A Tumor Organoid Model for Adjuvant Treatment Selection

**DOI:** 10.3390/bioengineering12101121

**Published:** 2025-10-19

**Authors:** Arushi Tripathy, Sunjong Ji, Habib Serhan, Reka Chakravarthy Raghunathan, Safiulla Syed, Visweswaran Ravijumar, Sunita Shankar, Dah-Luen Huang, Yazen Alomary, Yacoub Haydin, Tiffany Adam, Kelsey Wink, Nathan Clarke, Carl Koschmann, Nathan Merrill, Toshiro Hara, Sofia D. Merajver, Wajd N. Al-Holou

**Affiliations:** 1 Department of Neurosurgery, University of Michigan, Ann Arbor, MI 48109, USA; tripatha@med.umich.edu (A.T.); rekaragh@med.umich.edu (R.C.R.); safiulls@med.umich.edu (S.S.); sunitas@med.umich.edu (S.S.); dlhuang@med.umich.edu (D.-L.H.); yalomary@umich.edu (Y.A.); yhaydin@umich.edu (Y.H.); hara@umich.edu (T.H.); 2 Division of Hematology and Oncology, Department of Pediatrics, University of Michigan, Ann Arbor, MI 48109, USA; jsunjong@med.umich.edu (S.J.); tiffadam@umich.edu (T.A.); winkk@umich.edu (K.W.); ckoschma@med.umich.edu (C.K.); 3 Division of Hematology and Oncology, Department of Internal Medicine, University of Michigan, Ann Arbor, MI 48109, USA; haserhan@med.umich.edu (H.S.); nmerrill@med.umich.edu (N.M.); smerajve@med.umich.edu (S.D.M.); 4 Department of Computational Medicine and Bioinformatics, University of Michigan, Ann Arbor, MI 48109, USA; vravik@umich.edu; 5 Division of Neuro-Oncology, Department of Neurology, University of Michigan, Ann Arbor, MI 48109, USA; clarkena@med.umich.edu

**Keywords:** drug screening, H3K27M mutation, high-grade glioma, IDH-mutant glioma, patient-derived models, personalized medicine, precision oncology, spatial heterogeneity, translational bioengineering, tumor organoids

## Abstract

High-grade gliomas (HGGs) are aggressive brain tumors with limited treatment options and poor survival outcomes. Variants including isocitrate dehydrogenase (IDH)-wildtype, IDH-mutant, and histone 3 lysine to methionine substitution (H3K27M)-mutant subtypes demonstrate considerable tumor heterogeneity at the genetic, cellular, and microenvironmental levels. This presents a major barrier to the development of reliable models that recapitulate tumor heterogeneity, allowing for the development of effective therapies. Glioma tumor organoids (GTOs) have emerged as a promising model, offering a balance between biological relevance and practical scalability for precision medicine. In this study, we present a refined methodology for generating three-dimensional, multiregional, patient-derived GTOs across a spectrum of glioma subtypes (including primary and recurrent tumors) while preserving the transcriptomic and phenotypic heterogeneity of their source tumors. We demonstrate the feasibility of a high-throughput drug-screening platform to nominate multi-drug regimens, finding marked variability in drug response, not only between patients and tumor types, but also across regions within the tumor. These findings underscore the critical impact of spatial heterogeneity on therapeutic sensitivity and suggest that multiregional sampling is critical for adequate glioma model development and drug discovery. Finally, regional differential drug responses suggest that multi-agent drug therapy may provide better comprehensive oncologic control and highlight the potential of multiregional GTOs as a clinically actionable tool for personalized treatment strategies in HGG.

## 1. Introduction

High-grade gliomas (HGG) remain incurable; every tumor recurs following standard of care treatment with surgical resection, fractionated radiation therapy and temozolomide (TMZ) chemotherapy, with a median overall survival of approximately 12 months in IDH-wildtype (glioblastoma) and 24 months in IDH-mutant grade 4 astrocytomas [1,2,3]. Despite extensive research, effective treatment strategies are limited, in large part due to the profound heterogeneity of HGG at the genetic, cellular, and microenvironmental levels [4,5]. This intra-tumoral heterogeneity contradicts the development of universally effective therapies and underscores the need for models and multi-modal treatments that accurately reflect the complexity of the disease.

To model a heterogeneous tumor, progressively complex HGG models have been developed, from adherent cell lines and three-dimensional (3D) spheroids [6,7,8] to glioma tumor organoids (GTOs) and murine patient-derived xenografts (PDXs). While PDXs are highly faithful to patient tumors, they are time-consuming to establish and are not practical for immediate clinically applicable testing [6]. Conversely, GTOs provide a promising balance between histopathological, genetic, and phenotypic fidelity of the original tumor and rapid, scalable utilization for precision medicine applications [7,8,9,10,11].

Several methods have been employed to generate GTOs, each with its own advantages and limitations. Glioma-like features can be modeled through genetic manipulation of induced pluripotent stem cell (iPSC)-derived brain organoids, offering a platform for studying specific mutations, though these lack patient-specific tumor microenvironments [12]. Another approach involves bio-printing or fusion of malignant cells with non-malignant brain organoids to recreate tumor–niche interactions, though scalability and standardization remain technical hurdles [13,14,15]. Finally, organoids can be derived directly from resected patient tumors, preserving patient-specific features, including spatial heterogeneity and tumor microenvironment [16]. While tumor explants exclude the immune, vascular, and perfusion parameters of the other GTO models, their constitution is most similar to the original patient tumor [17,18,19,20].

Although GTOs have demonstrated capacity for testing targeted therapies and informing personalized treatment strategies, they are not yet routinely implemented in clinical precision medicine workflows [21,22,23,24]. Organoid-based drug testing has been shown in other cancers to have a strong correlation between ex vivo, in vivo, and patient responses [25]. Our group has developed a system for high-throughput drug testing in organoids, which has been successfully utilized to identify potential therapeutic biomarkers in bladder and breast cancer [26,27,28]. In this study, we extend this concept to glioma, incorporating a drug panel including pharmacotherapies covering known glioma oncogenic drivers and utilizing regionally sampled tumor tissues to explore the effect of spatial heterogeneity on drug response.

Additionally, we establish the feasibility of developing three-dimensional multi-focal patient-derived GTOs representing different subtypes of gliomas, including primary and recurrent tumors, IDH-wildtype glioblastoma, H3K27M-mutant, and IDH-mutant tumors. We demonstrate the ability of this model to recapitulate both tumor heterogeneity and clinical tumor genetic characteristics, capture both inter- and intra-tumoral heterogeneity, and importantly, the ability to be utilized for the development of rapid clinically relevant drug screening. Notably, we observed considerable variation in drug sensitivity by patient, by tumor type, and by intra-tumoral region, which has profound implications for surgical sampling, model generation, and importantly, for the development of clinically effective therapeutic regimens.

## 2. Materials and Methods

### 2.1. Collection of Glioma Tumor Specimens

**Figure 1:**
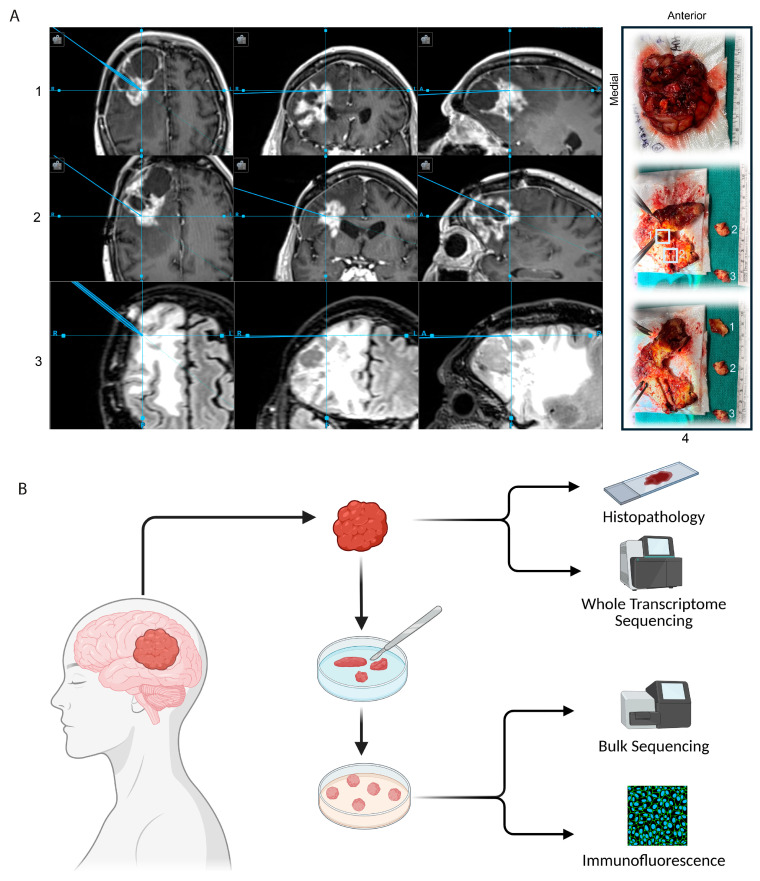

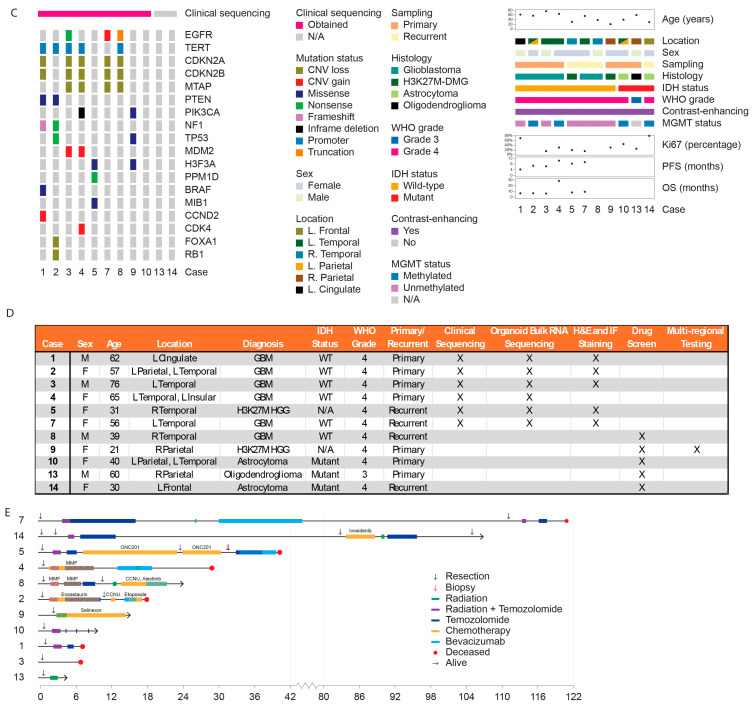
Tissue acquisition for GTO development: (**A**) Images demonstrating an example of stereotactic guidance to select tumor specimens for organoid production. Panels A1, A2, and A3 demonstrate portions of tumor selected for sampling. A1: enhancing 1 (anterior), A2: enhancing 2 (posterior), and A3: FLAIR. In panel A4, we demonstrate that the cystic portion of the tumor was decompressed, and the tumor removed en bloc. The explanted tumor was split open ex vivo, and the surgeon sampled visually non-necrotic tissue correlating with regions identified via stereotactic imaging. Radiographic regions corresponding with panels 1, 2, and 3 are, respectively, labeled in panel 4; (**B**) glioma tumor specimens were collected during clinically indicated tumor resection surgery—a portion underwent traditional histopathologic analysis in our institutional clinical laboratory, and a portion was prepared and sent for whole transcriptomic sequencing at Tempus (Chicago, IL, USA). The remaining portion was manually minced and cultured in ultra-low attachment plates on a rotary shaker until rounded organoids approaching 1.5 mm diameter in size formed. Organoids were analyzed via bulk RNA sequencing as well as grossly via fixation and fluorescence microscopy. (**C**) Tempus sequencing results and retrospective chart review were utilized to generate a summary of 11 patients included in this study. Patient 6, in whom clinical tumor sequencing was not ultimately submitted, was excluded, as were Patients 11 and 12, whose samples did not form an adequate quantity of organoids for screening. We present an oncoplot summary of clinical sequencing results, demographic information, histopathologic diagnostic analysis, and survival, demonstrating a wide range of tumor types and patient outcomes. (**D**) Table summarizing patient characteristics including demographics, diagnosis, and modality used during this study (clinical sequencing and bulk RNA comparison) or drug screening. (**E**) Swimmer’s plot summary of treatment course and outcomes of the same 11 patients. *X*-axis denotes months, *Y*-axis denotes clinical case reference number.

Brain tumor specimens were collected during clinically indicated tumor resection surgery at the University of Michigan with written informed consent under protocols #HUM00175135 and #HUM00024610, approved by the Institutional Review Board. Specimens were obtained intraoperatively during tumor resection by a brain tumor surgeon (author W.N.A.) utilizing stereotactic neuro-navigation (Stryker Q Navigation System) based on pre-operative Magnetic Resonance Imaging (MRI). Navigation was utilized to select samples corresponding with contrast-enhancing regions, fluid-attenuated inversion recovery (FLAIR) hyperintense regions, and regions considered to be along the brain–tumor edge (Figure 1A).

### 2.2. Clinical Analysis and Transcriptomic Sequencing

Tumor resection specimens were sent to the Clinical Pathology Department at the University of Michigan for fixation and paraffin embedding and underwent standard clinical histopathologic analysis (for pathologic diagnosis including staining of standard glioma markers such as Ki67—antigen Kiel 67—for proliferation index [29] and IDH, as well as methylation testing for O6-Methylguanine-DNA methyltransferase enzyme (MGMT), which is commonly tested for predicting the effects of alkylating chemotherapeutic agents). Tissue was also sent for Tempus xR whole transcriptome genomic sequence analysis, which utilizes the Illumina Novaseq platform to report clinically relevant fusions and splicing events (Tempus AI, Inc., Chicago, IL, USA) [30]. The Tempus xR test has been certified by CLIA (Clinical Laboratory Improvement Amendments) and accredited by the CAP (College of American Pathologists) federal regulatory institutions (Figure 1B). Whole exome and whole transcriptome sequencing data from Tempus were analyzed. Further clinical variables including survival, treatment course, radiographic characteristics, and clinical histopathologic diagnostics were manually collected via retrospective chart review by a licensed medical provider (author A.T.) and reviewed by the primary surgeon (author W.N.A), as approved under University of Michigan Institutional Review Board protocol #HUM00175089 (Figure 1C,D).

### 2.3. Generation and Maintenance of Tumor Organoids

Tissue explants were immediately placed in RPMI-1640 (Gibco, 11875093, Waltham, MA, USA) in airtight conical tubes and immediately transported on ice to the laboratory (which is a 15 min walk from the operative theater) by author D.H. Then, they were manually minced using a Number 10 blade and washed in phosphate-buffered saline (PBS, Arlington, VA, USA) (to remove blood, plasma, and tissue debris). Minced explants were placed in ultra-low attachment 6-well plates (Corning #3471, Corning, NY, USA), suspended in 4 mL per well of Tumor Organoid Media (a 1:1 combination of DMEM F12 (Gibco 11320033) and Neurobasal media (Gibco 21103049) supplemented with 5 mL antibiotic-antimycotic solution (Gibco, 15240062), 5 mL Glutamax (Gibco 35050061), 5 mL N_2_ supplement (Gibco A1370701), 10 mL B27 without vitamin, (Gibco 12587010), 5 mL Non-Essential Amino Acids (Gibco 11140050), 400 μL insulin (Gibco 12585014), and 500 μL 2-mercaptoethanol (Gibco 21985023)) on a rotary shaker in an incubator at 37 degrees Celsius and 5% carbon dioxide (Figure 1B). Organoid culture in media has been previously demonstrated to maintain superior faithfulness to patient-derived tissue characteristics overgrowth in synthetic extracellular matrix [31].

Three-fourths of the media was replaced twice weekly for 2 to 4 weeks until rounded organoids formed. Once organoid size approached 1.5 mm, some organoids were disrupted using AccuMax^TM^ (Stem Cell Technologies 07921, Vancouver, Canada), and viability assessed via trypan blue staining (Gibco 15250061) and a Countess 3.0 Cell Counter (Invitrogen, Waltham, MA, USA).

### 2.4. Organoid Fixation, Sectioning, and Slide Preparation

Organoids were manually individually placed into 500 μL Eppendorf tubes and embedded in a drop of Histogel (Epredia™ HG-4000-012, Portsmouth, NH, USA). This was allowed to completely harden prior to fixation in formalin for 12 h overnight (as previously demonstrated [32,33]), and embedding in paraffin blocks. Five-micron slices were cut via microtome and mounted onto slides.

Formalin-fixed, paraffin-embedded (FFPE) tissue sections were deparaffinized and rehydrated through a graded series of solvents. Slides were first immersed in 100% Xylenes (Fisher Chemical, LC269704, Waltham, MA, USA) for 3 min, followed by a second incubation in fresh 100% xylene for 1 min to ensure complete removal of paraffin. The sections were then passed through a descending ethanol series of 1 min incubation each to rehydrate the tissue: 100%, 95%, 85%, and 70% ethanol. Finally, slides were rinsed in 100% distilled water for 1 min to complete the rehydration process.

### 2.5. Hematoxylin and Eosin Staining

Following rehydration, tissue sections were stained using standard hematoxylin and eosin (H&E) staining protocol. Slides were incubated in Hematoxylin (Sigma-Aldrich GHS332, St. Louis, MO, USA) for 45 s, followed by a rinse in distilled water until the hematoxylin stain visibly faded, indicating removal of excess dye. Sections were then immersed in 95% ethanol for 1 min before staining in Eosin Y 0.5% (Sigma-Aldrich 41116124) for 1 min and 20 s. After eosin staining, the slides were sequentially dehydrated through increasing ethanol concentrations for 1 min each: 70%, 85%, 95%, and 100% ethanol. The slides were then cleared in 100% xylene for 1 min. Coverslips were mounted using Permount™ Mounting Medium (Fisher Chemical™ SP15-100). Slides were imaged using standard light microscopy.

### 2.6. Immunofluorescence Staining

Antigen unmasking was performed using Diva Decloaker (Biocare Medical DV2004, Pacheco, CA, USA) and 10 min of microwave heating as previously described [34]. Slides were washed in PBST (PBS (Gibco 10010023) with 0.1% Tween-20 (Sigma Aldrich P1379)) and quenched with 3% hydrogen peroxide for 10 min before proceeding with blocking. Slides were blocked with 5% Donkey serum (Sigma Aldrich D9663) diluted in PBST for 30 min at room temperature. Primary antibodies were diluted in 5% donkey serum at the following concentrations: Olig2 Rabbit (Abcam ab109186, Cambridge, UK) 1:100, Olig2 Goat (R&D Systems AF2418, Minneapolis, MN, USA) 10 μg/mL, CD44 Rat (Cell Signaling Technology #95235, Danvers, MA, USA) 1:100, and EGF Receptor XP Rabbit (Cell Signaling Technologies #4267) 1:25. Slides were incubated in primary antibody for 16 h, washed with PBST, and then incubated with secondary antibody for 60 min in the dark at room temperature. Secondary antibodies were diluted in 5% donkey serum at 1:1000 concentration (donkey anti-goat Alexa Fluor™ 647 Invitrogen™ A21447, donkey anti-rabbit DyLight™ 755 Invitrogen™ SA510043, donkey anti-mouse Alexa Fluor™ 488 Invitrogen™ A21202). Slides were washed again three times with PBST, allowed to dry, and cover slips were mounted onto the slides using VECTASHIELD Antifade Mounting Medium with DAPI (Vector Laboratories UX-93952-24, Newark, CA, USA). These were allowed to cure in the dark for at least 24 h at room temperature, and at 4 degrees Celsius for at least 24 additional hours prior to image acquisition on a Zeiss LSM 980 Airyscan 2 (Ziess, Oberkochen, Germany).

### 2.7. Organoid Bulk RNA Sequencing

Glioma tumor organoids were cultured and harvested as outlined above, and RNA was extracted using the RNeasy Mini Kit (Qiagen 74104, Venlo, The Netherlands) according to the manufacturer’s protocol. Initial quality control was performed using Qubit (Invitrogen Q33238) to determine concentration and Tapestation (Agilent 5067-5576, Santa Clara, CA, USA, TapeStation Analysis Software 4.1.1) for an RNA integrity number (RIN) quality score. Libraries were prepared using the NEBNext Ultra II RNA Library Prep Kit (New England Biolabs, Ipswich, MA, USA) with poly(A) selection. The samples were subjected to 151 bp paired-end sequencing according to the manufacturer’s protocol (Illumina NovaSeqXPlus, System Suite Version: 1.3.0.39308). BCL Convert Conversion Software v4.3.13 (Illumina, San Diego, CA, USA) was used to generate de-multiplexed fastq files. Data was processed by the Advanced Genomics Core at the University of Michigan.

### 2.8. Bulk RNA Sequencing Analysis

To infer relative cell type and cell state abundances from bulk RNA-sequencing data, we used the deconvolution algorithm BayesPrism [35]. Since tumor cell states are labeled differently in IDH-WT versus H3K27M gliomas, we used corresponding single cell reference datasets from Ruiz-Moreno et al. [36] and Liu et al. [37], respectively. Activity scores for major signaling processes were computed using the PROGENy model in decoupleR [38]. Relative abundance of each cell type and cell state were displayed as heatmaps generated using GraphPad Prism Version 10.4.1.

### 2.9. High-Throughput Drug Testing

Organoids were dissociated using Accumax^TM^ (Stem Cell Technologies 07921) then counted using an Acridine Orange/Propidium Iodide Stain (Logos Biosystems F23001) and a LUNA-FL^TM^ Dual Fluorescence Cell Counter (Logos Biosystems, Gyeonggi-do, Republic of Korea). Cells were then seeded in 96-well U bottom plates (S-bio-MS-9096W) using Human Plasma-like Medium (HPLM) (Gibco A4899101) supplemented with 10% Dialyzed FBS (Gibco 26400-044), 1% Penicillin-Streptomycin (Gibco 15140-122), 0.05% Gentamicin (Gibco 15750-060), and 1% DL-2-Hydroxybutryic Acid Sodium Salt, 97+% (Thermo Scientific^TM^ A18636.06, Waltham, MA, USA). Cells were seeded at a density of 1000–3000 cells/well, depending on the number of cells available to screen. On day 0, drugs were serially diluted 5-fold across eight concentrations in Dimethyl Sulfoxide (DMSO) (Millipore Sigma D2650, Burlington, MA, USA) in a 96-well round conical-bottom plate (Thermo Scientific^TM^ 249946), except for WP1122, which was dissolved in water. Drugs were then diluted 10-fold into HPLM, then added to the cells in duplicates via a 100-fold dilution, achieving a 1000-fold dilution overall. Cells were then incubated at 37 °C and 10% CO_2_. On day 5 (or for DNA damage repair [DDR] inhibitors, day 10), cell viability was measured using 3D Cell-titer Glo (Promega G9683, Madison, WI, USA) on a BioTek Synergy H1 Multi-Mode Reader (Agilent SH1FSN).

Cell viabilities were normalized to vehicle control and plotted on GraphPad Prism 10.4.1 using a four-parameter non-linear regression model with the function Y = Bottom + (Top − Bottom)/(1 + 10Log10(IC50) − X), where Y = Relative Viability and X = Log10(Molar Concentration), constraining the curves to Bottom > 0 and Top = 100. IC50 was calculated by GraphPad Prism Version 10.4.1. and C_ave_ (average plasma concentration) and C_max_ (maximum plasma concentration) doses were obtained based on pharmacokinetic data from FDA drug approval documents or published phase I clinical trials. Viabilities at C_ave_ and C_max_ were obtained by extrapolation from the four-parameter non-linear regression dose–response curves, as surrogates for drug response [39]. Drug Sensitivity Score 3 (DSS3) was calculated using R-studio (Version: 2025.05.0) based on area under the curve of a dose–response curve, with 20 chosen as a cutoff value for a clinically active drug (0–9 was considered inactive, 9–20: low activity, 21–29: semi-active, 30–59: active, and >60: very active) [40].

The drugs tested on each sample were selected based on cell count (to determine number of drugs included in each panel) and informed physician choice (for agents included, considering FDA-approved, experimental, and clinical trial drugs suitable for tumor subtype and known mutational drivers based on early histopathologic diagnosis and rapid sequencing). All drugs were purchased from MedChemExpress (Monmouth Junction, NJ, USA) or Selleckchem (Houston, TX, USA).

## 3. Results

### 3.1. Generation of GTOs

We selected a 3D GTO generation protocol that utilizes direct tumor explants (which has previously been shown as the method that best maintains glioblastoma intra-tumoral architecture and diverse cellular composition [9]) in order to most effectively recapitulate the treatment response of the patient-derived tumor (Figure 1B). We apply this technique to additional varieties of gliomas, including IDH-wildtype glioblastoma (which has been previously reported) as well as H3K27M-mutant tumors and IDH-mutant glioma [7].

In each patient, tumor specimens (0.5–1 cm^3^) were chosen for GTO generation based on stereotactic navigation as well as surgeon expertise in choosing non-necrotic tissues with a likelihood of higher live-cell component (Figure 1A). Organoid formation varied according to patient-derived tumor type (per Clinical Pathology standardized diagnosis), with IDH-wildtype (WT) glioblastoma GTOs typically forming within two weeks, and IDH-mutant GTOs taking four or more weeks.

### 3.2. Cohort Clinical Characteristics

Our cohort included 11 patients with the following diagnoses: primary and recurrent IDH-WT glioblastoma, primary and recurrent IDH-mutant HGG, and primary and recurrent H3K27M-mutant HGG (Figure 1D). Most tumors were WHO grade 4, with one WHO grade 3 tumor included. Patient age ranged from 21 to 76 years, and 36% (n = 4) were male. Nine patients underwent sequencing of their clinical samples with Tempus^TM^ whole genome sequencing (Chicago, IL, USA), identifying mutations in EGFR (n = 3), TERT (n = 5), CDKN2A and CDKN2B (n = 5), MTAP (n = 4), PTEN (n = 2), PK2CA (n = 2), NF1 (n = 2), TP53 (n = 2), MDM2 (n = 2), H2F2A (n = 2), and others (Figure 1C). On clinical pathology and methylation testing, four tumors were MGMT-methylated and Ki67 index [29] ranged from 15% to 80%. Radiographically, all tumors possessed contrast-enhancing components and arose from a variety of origins including bilateral temporal and parietal lobes, cingulate gyrus, and the left frontal lobe.

Overall survival of patients ranged from approximately 6 to 70 months, during which time patients underwent surgery as well as courses of radiation, temozolomide, bevacizumab, and other chemotherapies. Five patients included in the study remain alive at the time of writing this manuscript (Figure 1E).

### 3.3. Characterizing Heterogeneity Between GTOs

**Figure 2:**
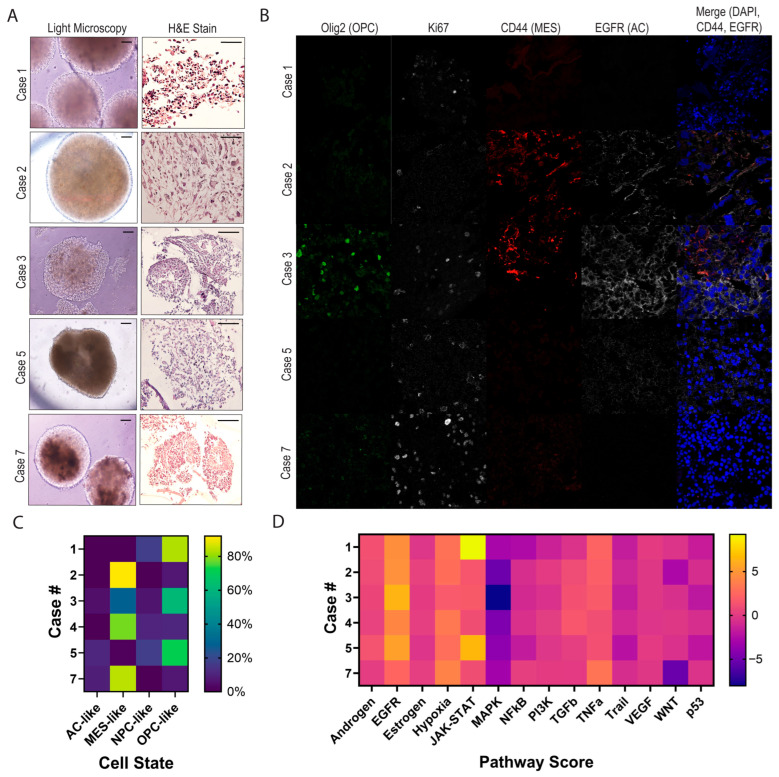
Characterization of GTOs: (**A**) Glioma tumor organoids were imaged at low magnification using light microscopy, demonstrating variation in gross composition and appearance (Scale bars, 200 μm). GTOs were individually fixed and stained with hematoxylin and eosin. Imaging at higher magnification using light microscopy demonstrates variation in cell morphology and gross architecture of extracellular matrix (Scale bars, 100 μm). Case 4 is excluded in H&E and IF stain figure panels (**A**,**B**) due to processing variability resulting in low density cellular imaging. (**B**) Variation in glioma cell state markers (as characterized by Neftel et al. [41]) is assessed via immunofluorescent staining using a 40× objective, staining for OLIG2 (green), Ki67 (white), CD44 (red), EGFR (white), and DAPI (blue). (**C**) Bulk RNA sequencing analysis of GTOs demonstrates a higher proportion of MES-like and OPC-like states among organoids (scale bar on the right denoting percentage as a measure of proportion). (**D**) GTO bulk RNA sequencing analysis also demonstrates variation in pathway activity across studied organoids (scale bar on the right denoting scale as a measure of relative activity).

In Cases 1–5 and 7, GTOs were analyzed via bulk RNA sequencing, histological sectioning, and immunohistochemistry/immunofluorescence (IHC/IF) (Figure 2). In order to observe the macro- and micro-architecture of the GTOs, they were first imaged using bright-field microscopy, which demonstrated gross variation in structure and heterogeneity across tumors (Figure 2A). Hematoxylin and eosin stains of these GTOs demonstrated intra- and inter-organoid heterogeneity in cell density, cell morphology (ranging from small anaplastic cells to pleomorphic giant cells and gemistocytic cells), and nuclear morphology (from normal to elongated), for example, Case 2 had especially large abnormally shaped nuclei, while Cases 1 and 7 had relatively uniform nuclear shape.

Gliomas have been characterized to express cells of four main cell states defined by transcriptomic signatures, as described by Neftel et al. [41] To assess the representation of these cell states among these GTOs, we utilized IHC/IF to observe compositional and spatial heterogeneity in cell state expression across tumors. Olig2, CD44, and EGFR antibodies were used to represent oligodendrocyte precursor cell-like (OPC-like), mesenchymal-like (MES-like), and astrocyte-like (AC-like) cell states, respectively. Cell state staining demonstrated prominent variation in expression of these markers between GTOs. Expression of Olig2, CD44, and EGFR were high in Case 3, while Case 2 mainly displayed prominent CD44 expression (Figure 2B). Organoids were further stained for Ki67 and were similarly found to express a range of proliferation indices, as expected from a heterogenous cohort (Figure 2B). The organoids derived from Case 7 had the highest staining for Ki67 via IF; however, clinical tumor staining demonstrated a lower Ki67 index. Similarly, the Case 1 organoids had low Ki67 staining (Figure 2B), while clinical patient-derived tumor stains demonstrated high Ki67 index (Figure 1C).

We then evaluated the transcriptomic signatures of GTOs. Deconvolution of the tumor organoids was performed using single cell reference datasets to estimate relative cell state abundance using marker sets delineated by Neftel et al. [28] and Liu et al. (for H3K27M tumors) [29]. Our findings demonstrate profound variation in cell state representation across the GTO cohort, differentiating two main categories of GTOs—primarily OPC-like and primarily MES-like (Figure 2C).

Comparing cell state marker expression via IF versus bulk RNA sequencing, we find that in some cases the findings are aligned (e.g., Case 2 demonstrates predominantly MES-like marker CD44, and Case 3 demonstrates a mixture of OPC-like marker Olig2 and MES-like marker CD44) (Figure 2B,C). However, in others, cell state marker proportions did not match between IF and the transcriptome (e.g., Case 1 did not have appreciable OPC-like Olig2 expression via IF, Case 7 did not have high MES-like CD44 expression) (Figure 2B,C). As cell states are delineated based on relative expression of a set of markers, a single marker is likely an imperfect surrogate for estimating each cell’s comprehensive transcriptional activity; thus, this discrepancy is within expectations.

We further analyzed GTO RNA bulk sequencing data according to pathway analyses, demonstrating considerable inter-GTO heterogeneity in transcriptional activity of WNT, MAPK, JAK-STAT, hypoxia, and EGFR pathways (Figure 2D). Androgen, estrogen, and VEGF pathway enrichment was similar across all tumors. In Case 3, pathway scores mirrored strong IF EGFR signals (Figure 2B,D).

### 3.4. GTOs May Recapitulate Patient Tumor Characteristics

**Figure 3:**
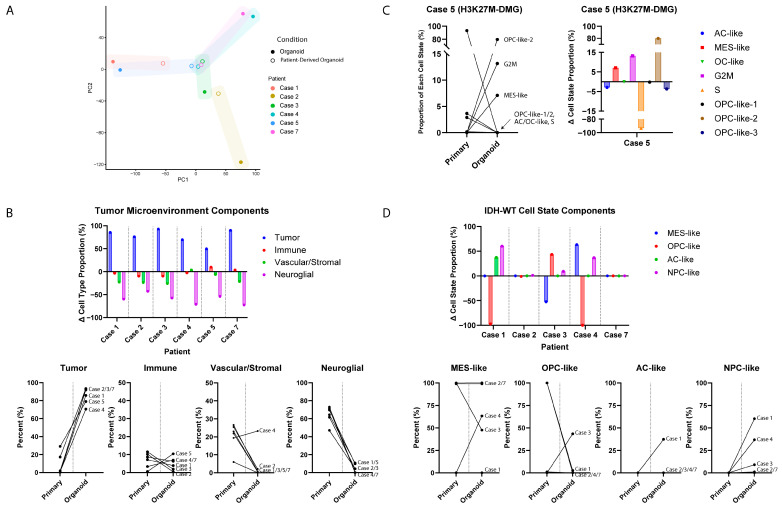
Comparative bulk sequencing analysis—GTO versus native tumor: (**A**) Tempus^TM^ whole transcriptome sequencing of patient-derived tumor samples were compared against bulk RNA sequencing of organoids derived from those tumors. Results are displayed in PC plot format, demonstrating that GTOs more closely colocalize with their paired patient tumor compared to other tumors; (**B**) deconvolution was used to identify cell type proportions within each organoid. While most organoids largely consisted of tumor cells, many models also demonstrated immune, vascular/stromal, and neuroglial components. *Y*-axis represents change in percentage from patient-derived tumor to GTO model; (**C**) cell state deconvolution analysis of H3K27M-DMG tumor sample and paired organoid model revealed a shift from an S-phase-dominated patient tumor to an OPC-like-2-dominant profile in the organoid. (**D**) Cell state deconvolution analysis of IDH-WT patient-derived tumor and matched organoid models demonstrating shift toward AC-like and NPC-like behavior in the organoids.

To compare organoids with their patient-derived tumors, we compared the results of whole transcriptome analysis of paired GTO and the original tumor samples. Principal component analysis (PCA) demonstrates that GTOs more closely colocalize with their paired patient tumor, as compared to other tumors (Figure 3A).

The sequencing datasets were analyzed using deconvolution to identify cell type proportions within each organoid and compared to the original patient-derived tumor. Comparatively, glioma organoids were composed of a greater tumor cell component versus the patient-derived tumor (mean 84.1% versus 6.5%, respectively). Although the neuroglial and vascular/stromal compartments were present in the GTOs, they were relatively depleted compared to the patient-derived tumor (mean −62.9 pp and −14.3 pp, respectively). Immune cell content remained low, with minor changes in composition between the matched tumors and organoids (mean 6.3% versus 5.9%; mean −0.4 pp) (Figure 3B).

Cell state deconvolution analysis of the H3K27M-DMG patient-derived sample and matched organoid model (Case 5) was performed utilizing states previously outlined by Filbin et al. [42]. This revealed a shift from an S-phase-dominated patient tumor (93.25%) to an OPC-like-2-dominant profile (79.74%) with some G2M (13.16%) and MES-like glioma cell states in the organoid (7.11%; OPC-like-2 +79.74 pp, G2M +13.16 pp, S −93.25 pp). After excluding cycling programs, the organoid was 91.8% OPC-like-2, whereas the original tumor’s cell state distribution was split between OPC-like-3 (54.4%) and AC-like (42.8%) (Figure 3C).

After restricting for tumor cell programs only, patient-derived tumors and organoids from all four IDH-WT pairs were dominated by canonical Neftel states. One pair (Case 7) showed near-identical MES composition between patient-derived tumor and matched organoid (+0.06 pp for MES-like; −0.06 pp for AC-like). Moreover, in the remaining pairs (Cases 1, 2, and 3), patient-derived tumors shifted from single-state primaries to bi-modal mixtures (Case 3 from MES-like-dominant to MES-/OPC-like; Case 1 from OPC-like-dominant to AC-/NPC-like; and Case 4 from OPC-like-dominant to MES-/NPC-like), with overall changes in composition ranging from −52.4 pp in Case 3 to −99.9 pp in Case 4. Of note, the patient-derived tumors for these matched pairs had relatively lower tumor content, demonstrating the sustained capacity of their matched tumor organoids to recapitulate other glioma cell states (Figure 3D).

### 3.5. Heterogeneous Drug Reponses Between GTOs

**Figure 4:**
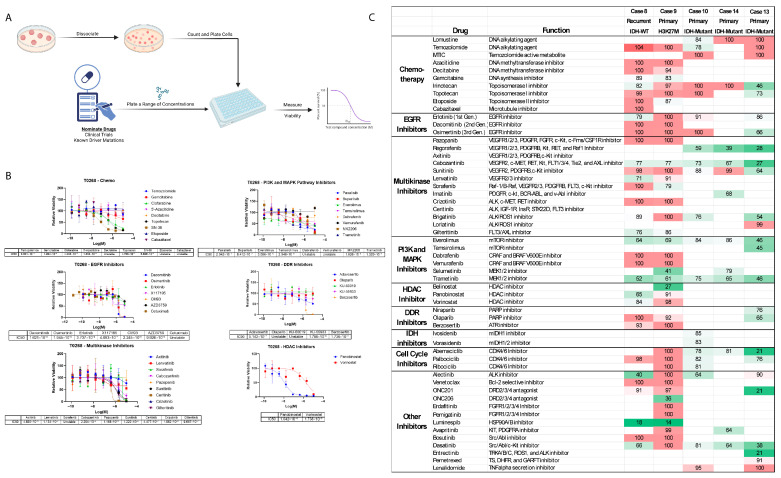
Drug screen: (**A**) Workflow: organoids were dissociated into single cell suspension, counted, and plated at uniform density. A panel of drugs was chosen based on driver mutations (identified via clinical histologic diagnostic staining, and non-clinical laboratory rapid sequencing using the NanoPore platform [Oxford, UK]), and clinical trial availability. Each drug was tested across a range of concentrations and viability was assessed at 5 days. Finally, IC50 values were generated based on viability curves. (**B**) Calculation of IC50: each organoid culture was dissociated and cells counted—in each screen, the panel of drugs was chosen using known targetable mutations where applicable and limited by cell count as indicated. In each screen, several drug classes were included and tested at a range of concentrations. At 5 days (10 for DDR inhibitors) cell viabilities were plotted and IC50 values calculated for each drug. Each datapoint represents an average between assays completed in duplicate. The viability curves and IC50 calculations for Case 8 are demonstrated here as an example. (**C**) A comprehensive summary of cell viability (in percentage) at C_ave_ (average plasma concentration) following 5 days of treatment across all tested organoids and all included drugs in each screen. The table is color coded with progressively darker green shades denoting increasing drug effectiveness in reducing cell viability.

In five additional patients, GTOs were utilized for high-throughput drug screening (Cases 8–10, 13, and 14). To assess GTO response to various drugs, we optimized a high-throughput drug screening workflow and performed drug screening on a subset of organoids spanning tumors characterized as glioblastoma (IDH-WT), high-grade astrocytoma (IDH-mutant), oligosarcoma (IDH-mutant), and H3K27M hemispheric glioma. Organoids were maintained preserving tumoral architecture (and thereby preserving intercellular interactions, transcriptional activity, and protein expression) until immediately prior to the drug screening (Figure 4A). Drugs were each tested at a range of concentrations to calculate IC50 and determine viability at C_max_ and C_ave_ (Figure 4B). A variety of drug types were included, ranging from chemotherapeutics to specific and multi-kinase inhibitors, pathway inhibitors, DNA damage repair (DDR) inhibitors, and histone deacetylase (HDAC) inhibitors (Figure 4C).

**Figure 5:**
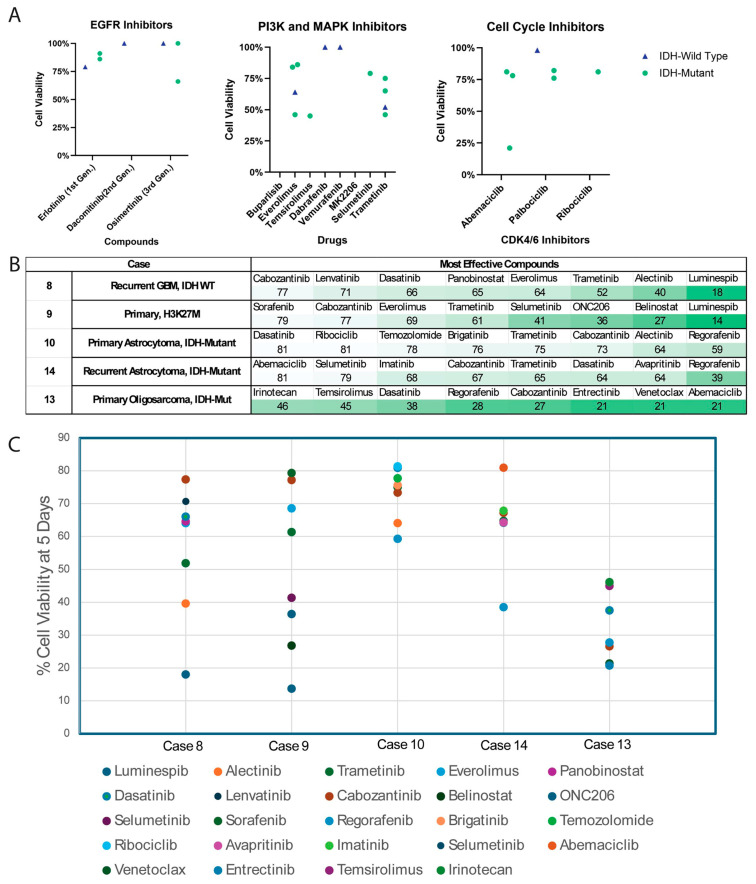
Selected drug screening results: (**A**) responses of IDH-WT and IDH-mutant to EGFR inhibitors, PI3K/MAPK inhibitors, and cell cycle inhibitors. There does not appear to be a significant superiority of any one drug class for WT or mutant tumors. (**B**,**C**) The eight most effective drugs are listed for each tumor, with percentage cell viability listed following 5 days of treatment in vitro for each drug. Progressively darker shades of green denote higher effectiveness.

As expected, due to profound inter- and intra-tumoral heterogeneity among glioma, no single agent or drug target proved to be effective against all five tumors (Figure 4C). EGFR inhibitors, PI3K and MAPK inhibitors, and cell cycle inhibitors generally had similar effects in IDH-mutant versus IDH-WT tumors (Figure 5A). When the top eight drugs with the highest effectiveness against a tumor were ranked, both IDH-mutant tumors were highly responsive to regorafenib and cabozantinib, while the IDH-WT and H3K27M-mutant tumors were responsive to luminespib and trametinib (Figure 5B,C). IDH-mutant astrocytomas were more resistant to drugs compared to IDH-mutant oligosarcoma, with smaller decreases in cell viability overall (Cases 10 and 14, Figure 5C).

### 3.6. Multiregional GTO Drug Testing Demonstrates Heterogenous Response

**Figure 6:**
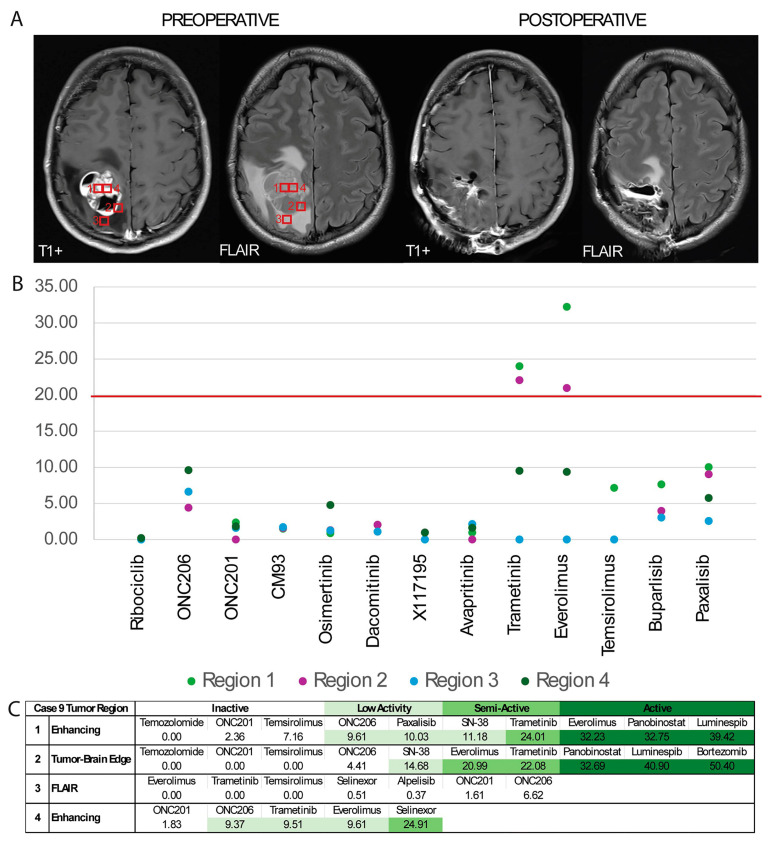
Case 9 multiregional drug screen: (**A**) T1+ (contrast-enhancing) and FLAIR MRI sequences with sampled regions labeled. Screening was initially completed for regions 1 (contrast-enhancing) and 2 (tumor–brain edge) and subsequently completed for regions 3 (FLAIR) and 4 (contrast-enhancing); (**B**) DSS3 scores were computed to determine predicted clinical activity of each drug, with clinical activity threshold traditionally set at 20 (horizontal line). All drugs tested across at least three regions are presented. Everolimus and trametinib achieve clinical activity in regions 1 and 2 however do not in regions 3 and 4; (**C**) drugs found to have clinical activity (according to DSS3 score [32]) or clinical relevance (according to PI3K targeting or clinical trial availability) are included rated by region, and color-coded according to activity: <9 inactive, 9–20: low activity, 21–29: semi-active, and 30–59: active). Panobinostat, luminespib, and bortezomib were not tested in regions 3 and 4. Color code is described in Figure 5C description.

To determine whether spatial tumor heterogeneity would alter drug response, in one patient—Case 9—with an H3K27M-mutant tumor, the lesion was sampled using stereotactic guidance for organoid generation from three distinct radiographically defined regions: FLAIR-hyperintense, contrast-enhancing, and tumor–brain edge (Figure 6A). As the infiltrating tumor is sometimes left behind during surgical resection, we aimed to identify potential differential drug responses between tumor regions that are typically resected and regions that are commonly not resected.

Two of the tissues—contrast-enhancing and tumor–brain edge—readily expanded in organoid culture and drug screening was completed in our usual timeline. The FLAIR-hyperintense tissue was slow-growing and thus cultured for an additional week prior to drug testing. During this week, rapid sequencing of the patient’s tumor was completed, identifying H3K27M and PIK3CA mutations. Given that a smaller quantity of organoids had grown from the FLAIR culture, a shortened altered panel of drugs was used for third and fourth drug screens for this patient (the FLAIR organoids, and an additional contrast-enhancing culture adjacent to that of the initial drug screen).

We determined that different portions of the tumor demonstrated variability in drug response, with clinically semi-active and active DSS3 [32] scores for trametinib (a MEK 1/2 inhibitor) and everolimus (an mTOR inhibitor) in the first contrast-enhancing organoids and the tumor–brain interface. However, there was no response to these two agents in the FLAIR organoids, and low activity of these agents in the second contrast-enhancing organoids (Figure 6B,C). Neither FLAIR nor contrast-enhancing organoids were responsive to ONC201; however, Selinexor appeared to have some activity against the contrast-enhancing organoids (Figure 6B,C).

Generally, H3K27M tumors have a poor prognosis, with a median survival of 9–12 months with treatment. In this case, after resection and standard of care radiation, the patient was eligible for clinical trials at our institution and was enrolled in a Selinexor trial and has not shown evidence of progression at the time of writing this manuscript (patient has been maintained for 13 months on the drug, beginning one month following resection). This clinical result remarkably demonstrates consistency with the results of our GTO screening.

## 4. Discussion

In this study, we have developed patient-derived 3D GTOs, utilizing a technique previously described for glioblastoma [7], to reliably produce viable GTOs from a wide variety of gliomas, both primary and recurrent, with vast heterogenous spatial characteristics (Figure 1C). We demonstrate that these GTOs recapitulate significant inter-tumoral heterogeneity in glioma cell state proportions and tumor pathway activation (Figure 2C,D), and demonstrate their utility in rapid drug screening and for drug selection for patients with lethal HGGs.

All tumor models have advantages and disadvantages to their utilization. In this case, we felt that GTOs provided a rapid method to model patient-derived tumors and allow for rapid drug screening. In comparison, PDX models, which include a circulatory system for continuous nutrient and oxygen delivery, have several downsides. With some variation in individual clinical practice, following surgical resection, patients are traditionally initiated on adjuvant therapy as early as 2–6 weeks postoperatively (to allow for a minimum period for incisional healing prior to chemotherapy and radiation). PDX models can take weeks to months to develop, not all tumors reliably grow in vivo immediately, and have significant challenges and costs when considering drug screening. In comparison, 3D GTOs have many advantages. They contain a tumor microenvironment early on, and they have high likelihood of model formation typically within 2–3 weeks, which allows for rapid drug screening, which takes 5 days in our laboratory. Furthermore, drug screening of patient-derived organoids has been demonstrated to recapitulate responses in patients with certain systemic malignances [27,28]. Thus, GTOs provide an opportunity for the rapid development of patient-specific personalized treatment plans.

Employing parallel sequencing of patient tissues and organoids, we demonstrate that our organoids imitate the patient-derived tumors from which they are derived (Figure 3). However, we did appreciate some gene expression changes between the original tumor and its matched organoid. As expected, environmental pressures in cell culture will likely promote divergent expression of genes and shifts in tumor cell states. Furthermore, patient-derived tumors include a higher proportion of non-tumoral, microenvironmental cells (such as endothelial cells, myeloid cells, and astrocytes); thus, bulk RNA sequencing of these tumor explants will generally include genes expressed by the host environment. However, these other cell types typically do not survive well ex vivo in GTO culture. As a result, in some cases, the original patient-derived tumors were more similar to one another than their respective GTOs. However, this was to be expected, and it was clear that organoids more closely resembled their matched tumor than other tumors. Stressors of forced growth in culture may promote expression of unique oncogenes (Figure 3). When sequencing datasets were analyzed for cell type proportions, GTOs were composed of a greater tumor cell component compared to patient-derived tumor samples (Figure 3B). As expected for in vitro culture systems lacking tumor microenvironments, neuroglial and vascular/stromal compartments were depleted in glioma organoids. These data indicate that patient-derived glioma organoids enrich malignant cells while decreasing non-tumor compartments over time.

The H3K27M-DMG case in our cohort demonstrated a shift from an S-phase-dominated patient-derived tumor (93.25%) to an OPC-like-2-dominant GTO profile (79.74%). These changes support that the GTO model recapitulates the malignant OPC-like program typical of H3K27M-DMG while appropriately minimizing non-tumor components (Figure 3C). GTOs from all four IDH-WT pairs demonstrated strong enrichment in previously defined cells states of patient-derived glioma tissues [32,37]. These data support that patient-derived organoids not only are able to model glioma cell intrinsic transcriptional programs but also model the plasticity essential for glioma’s invasive behavior (Figure 3D).

GTOs initially faithfully recapitulate the tumor microenvironment, including proportions of cell type, cell state, as well as spatial heterogeneity. Over time, without a viable circulatory system and tumor microenvironmental signaling factors, this heterogeneity is slowly lost, as previously described [7]. Thus, though we maintain nearly 120 organoid cultures at any given time, with some as old as 1.3 years old, we limited drug testing to organoids generated within a couple weeks of resection. We plan to further study organoid evolution over time in response to drug therapy as we scale this platform.

### 4.1. Production of IDH-Mutant GTOs

Multiple models have been generated for the study of glioblastoma (IDH-WT), ranging from adherent cell lines (which are easy to maintain but fail to represent intercellular interactions essential to glioma growth and behavior) to PDX models (which are time-consuming to generate and laborious to maintain, and have a limited lifespan of study). However, IDH-mutant cell lines and organoids are less frequently successfully generated [43,44,45,46]. In our experience, while generation of IDH-mutant GTOs is feasible, their growth is slower compared to IDH-WT gliomas, with a lower success rate closer to 80%, with a varied and prolonged generation time ranging from 3 weeks to 2 months. In this study, two of the five initially included IDH-mutant cases (Cases 11 and 12) did not grow sufficient organoids for drug screening and were excluded. There appears to be a technical challenge in producing IDH-mutant cell lines and organoids due to inherent slower growth and thus diminished capacity for testing. IDH-mutant tumors have an extended clinical timeline compared with glioblastoma (IDH-WT); therefore, an inability to produce testable organoids within 2 weeks for IDH-mutant tumors may not be clinically problematic. A slower timeline of organoid generation, growth, and testing may be acceptable in IDH-mutant tumors.

While previous methods have demonstrated the feasibility of drug testing in glioblastoma, no such model has been generated and tested for IDH-mutant tumors [14]. Nonetheless, in this study we demonstrate that it is not only possible to produce IDH-mutant and H3K27M organoids, but they can also be generated with a frequency and volume necessary for a high-throughput, clinically relevant drug testing paradigm.

### 4.2. In Vitro GTO Drug Testing Results Match Clinical Response

Where available, drug screening results generally mirrored molecular testing results in patients. For example, O(6)-methylguanine-DNA methyltransferase (MGMT) is known to antagonize the effects of alkylating agents, and MGMT methylation status has been used to predict glioma response to DNA alkylating agents [47]. Of the organoids tested in our study, alkylating agents TMZ and lomustine, as well as TMZ’s active metabolite MTIC, were found to be ineffective in Cases 8 and 9, which were derived from MGMT-unmethylated tumors. As expected in Case 10, the MGMT-methylated tumor was sensitive to alkylating agents TMZ and lomustine in vitro (Figure 1C and Figure 4C).

Vorasidinib has been demonstrated to have efficacy in low-grade recurrent or residual IDH-mutant glioma, and ivosidenib has been shown to reduce growth and improve control in non-enhancing IDH-mutant glioma [48,49]. An IDH-mutant tumor in our cohort that was screened against IDH-inhibitors ivosidenib and vorasidinib predictably demonstrated modest responsiveness to both agents. The other IDH-mutant astrocytoma (Case 14) was not screened for IDH inhibitors; however, it had previously received ivosidenib therapy (Figure 5C).

The patient-derived tumor in Case 8 was found to have EGFR copy number gain and did demonstrate some response to the EGFR inhibitor erlotinib (Figure 5A). Finally, selinexor appeared to have some activity against the contrast-enhancing GTOs in Case 9, and the patient treated with this drug has remained progression-free for 13 months (Figure 6B,C). The expected response of tumors possessing well-studied mutations to well-studied agents provides a foundation for validity of GTO response to newly tested drugs in our high-throughput platform.

### 4.3. Strategies for Utilizing Drug Testing Results

High-throughput drug testing may help screen drugs to nominate a drug for further study. Trial of a multitude of drugs against numerous tumors could be expected to elucidate a monotherapy or drug class that is effective across many or most gliomas. However, in our preliminary approach studying five tumors, testing did not identify a single agent or drug class that preferentially treated IDH-WT or IDH-mutant gliomas as a whole (Figure 5A).

Testing can also be used to nominate multi-agent regimens for adjuvant therapy. Due to the inter-tumoral heterogeneity in glioma, we utilized the results of each drug screen to generate a list of the eight most effective drugs (Figure 5B,C). In these cases, a multidisciplinary team including neuro-oncologists, medical oncologists, and pharmacists could apply clinical knowledge of the ability of these agents to cross the blood–brain barrier, their side-effect profiles, and their cross reactivity to choose two or three agents to administer in tandem. Most cancers are approached with multi-drug regimens; thus, this multi-faceted approach may be more effective in targeting malignant gliomas. Ex vivo GTO drug screening may be utilized as a personalized eligibility criteria for more effective patient selection in these types of trials. For example, Case 8 with EGFR copy number gain was responsive to the EGFR inhibitor erlotinib; however, it was not responsive to dacomitinib or osimertinib (Figure 5A). The EGFR TKIs each have slightly different protein structures, allowing for irreversible binding of the drug to EGF receptors with differing mutations [50]. Personalized drug screening with organoids presents a viable method to determine tumor sensitivity to unique EGFR inhibitors without extensive personalized sequencing.

### 4.4. Multiregional Drug Testing Reveals Differential Results

It has been previously demonstrated that gross- or supra-total resection improves outcomes in glioma [51,52,53]. Given the infiltrative nature of glioma, adjuvant pharmacotherapy and radiotherapy are the standard of care for treatment of inevitable residual disease at the margins of the tumor. Preclinical patient-derived tumor models are generated from resected tumor samples; however, to our knowledge, these marginal edges are not commonly differentially studied in vitro at this time. Prior studies have demonstrated that interactions with normal brain extracellular matrix alter the tumor’s transcriptomic profile and potential drug response [19]. Thus, to determine whether resected and generally unresected (marginal) portions of the tumor demonstrate differential drug responsiveness, we performed multiregional drug testing, including organoids generated from contrast-enhancing, FLAIR, and brain–tumor edge samples.

Indeed, we did discover distinct drug activity between sampled regions—for example, everolimus demonstrated active DSS3 scores in the first contrast-enhancing sample, a semi-active score in the brain–tumor edge, low activity in the second contrast-enhancing sample, and no activity in the FLAIR sample (Figure 6B,C). Not only did radiographically distinct regions portend differential drug sensitivity, the two contrast-enhancing (radiographically identical) samples also demonstrated differential activity (significantly in trametinib and everolimus) (Figure 6B,C). These findings suggest that multiregional sampling may need to be considered for pathological/genetic clinical tumor analysis, as well as any preclinical studies that involve drug screening. This further supports that, given the intrinsic heterogeneity of gliomas, a multi-drug approach may be indicated to target the tumor comprehensively. Ultimately, our findings demonstrate that consideration of tissue sampling location is crucial when utilizing in vitro models for therapy development.

### 4.5. Limitations for Application of GTO Drug Screening

There are several limitations to this study. This is a small cohort of patients representing different subtypes of glioma. We demonstrate the ability to form organoids from different tumor types and show data suggesting that the organoids can recapitulate some features of the original tumors. However, given the environmental differences in cell culture compared to the original tumor, it is challenging to compare these matched tumors.

Furthermore, we suggest that these organoids may be used for drug screening. However, there are several limitations to this approach, and further validation is necessary. Although we demonstrate correlation between patient outcomes and genotype and organoid screening, further analysis and confirmation is necessary to affirm that this organoid screening platform can be utilized for clinical application. We intend for our drug screening results to allow clinicians to recommend multi-drug regimens based on C_ave_, C_max_, and DSS3. However, while these are all metrics suited for systemic drug bioavailability in plasma, brain tumors are unique in their immune-privileged location beyond the blood–brain barrier (BBB). Future window of opportunity trials (perhaps during surgical resection for recurrent tumors being treated with pharmacotherapies chosen via GTO drug screen) comparing plasma drug concentrations with tumor penetrance may inform drug screens. Radiation therapy is a contemporary mainstay of glioma treatment, and future drug screening trials may combine in vitro radiation and drug administration to account for cumulative effect and assess multiregional response to radiation [54]. Finally, immunotherapies have been increasingly studied in gliomas and may not be suitable for testing using this GTO platform.

Given the changing quantity of viable GTO cells for each drug screen in this study, a different drug panel was utilized for each of the five screens included. For more directed future study, we plan to abbreviate the number of drugs included in the panel so that more organoid models are eligible for screening and so that results are directly comparable.

Interestingly, in Case 10, GTOs (whose patient-derived tumor was MGMT-methylated) were responsive to TMZ; however, they were not responsive to MTIC, the active metabolite of TMZ (Figure 4C). MTIC is unstable in acidic media, so this finding may suggest that the media used for drug screening influences the results and should be controlled in the future [55,56].

## 5. Conclusions

This study establishes and validates a glioma tumor organoid platform capable of partially recapitulating the cellular, transcriptomic, and spatial heterogeneity of patient tumors, including IDH-mutant and H3K27M subtypes. By performing high-throughput drug screening on regionally sampled organoids, we reveal substantial inter- and intra-tumoral variability in drug response, highlighting the necessity of multiregional sampling to nominate therapies as well as predict response to therapy. Notably, we demonstrate that GTO-based drug testing might be useful and can inform clinical decision-making within a clinically actionable timeframe, offering a scalable and patient-specific approach to precision oncology in glioma which may improve outcomes and refine enrollment in clinical trials [57]. However, further validation is necessary.

## Data Availability

The original contributions presented in this study are included in the article. Further inquiries can be directed to the corresponding author. The raw data supporting the conclusions of this article will be made available by the authors on request.

## Abbreviations

The following abbreviations are used in this manuscript:

3D	Three-dimensional
BBB	Blood–Brain Barrier
Cave	Average Plasma Concentration
Cmax	Maximum Plasma Concentration
CAP	College of American Pathologists
CLIA	Clinical Laboratory Improvement Amendments
DMG	Diffuse Midline Glioma
DSS3	Drug Sensitivity Score 3
EGFR	Epidermal Growth Factor Receptor
FLAIR	Fluid-Attenuated Inversion Recovery
GBM	Glioblastoma Multiforme
GTO	Glioma Tumor Organoid
HGG	High-Grade Glioma
HPLM	Human Plasma-Like Medium
IHC/IF	Immunochemistry/Immunofluorescence
PDX	Patient-Derived Xenografts
RIN	RNA integrity number
TMZ	Temozolomide
WT	Wild Type
